# Manipulating Resistance Exercise Variables to Improve Jumps, Sprints, and Changes of Direction in Soccer: What We Know and What We Don’t Know

**DOI:** 10.3390/jfmk10020145

**Published:** 2025-04-26

**Authors:** Sandro Bartolomei, Marco Beato, Giuseppe Coratella

**Affiliations:** 1 Department of Life Quality Studies, Università Degli Studi di Bologna, 47921 Rimini, Italy; sandro.bartolomei@unibo.it; 2 School of Health and Sports Science, University of Suffolk, Ipswich IP4 1QJ, UK; m.beato@uos.ac.uk; 3 Department of Biomedical Sciences for Health, Università Degli Studi di Milano, 20133 Milan, Italy

**Keywords:** football, resistance training, strength, velocity-based training, velocity loss, range of movement, time under tension, inter-set rest, movement velocity, failure

## Abstract

The present review summarizes the effects of manipulating different resistance exercise variables on jumps, sprints, and changes of direction (CODs) in soccer. Regarding jumps, moderate-to-high loads, full range of movement (ROM), non-failure sets, and a moderate training volume are recommended. Different external resistances like constant-load, flywheel, or elastic bands, as well as various movement velocities and select exercises, are equally effective. As for sprints, moderate-to-high loads, constant load or flywheel but not elastic resistances, movements performed at full ROM, non-failure sets, and moderate-to-high training volume might be more effective, while numerous movement velocities and exercises could be chosen. As for CODs, moderate-to-high loads, flywheel more than constant-load resistance, and a moderate-to-high total number of repetitions are recommended, while several movement velocities and exercises could be selected, though ROM needs investigation. The effectiveness of concentric-only vs. eccentric-only training on jumps, sprints, and CODs has not been investigated, while an external focus and inter-set rest > 2 min are theoretically preferable, albeit not proven. Importantly, high movement velocity is not a prerogative of effectiveness, and limited ROM is not associated with sport-specific patterns such as jumps. Practitioners in soccer may manipulate resistance exercise variables depending on the purpose.

## 1. Introduction

Resistance training (RT) is an essential component of soccer conditioning aimed at enhancing performance [1,2], since research has shown that increases in muscular strength are associated with improvements in jump [3], sprinting [4], and changing of direction (COD) performance [5,6,7], all of which are crucial for soccer performance [8,9,10]. Interestingly, an increment in squat 1-RM of ~24% led to an increment in jumping ability of ~7%; ~20% is required to improve sprinting ability by ~2%, and ~15% to improve COD ability by ~1% [1]. Additionally, it should be recalled that plyometric training as a standalone modality or in combination with RT has been reported as effective in improving performance [11,12,13,14].

While the advantages of RT for soccer performance are evident, its implementation depends on several variables. Conventionally, RT exercises are scheduled as sets × repetitions × external load, even though such a historical approach appears too simplistic and overlooks many other variables that are part of any resistance exercise and affect the long-term adaptations, as already summarized for strength, hypertrophy, and muscle architecture [15,16,17]. Indeed, the organization of macro-, meso-, and micro-cycle resistance training refers to multiple independent parameters related to the organization of the session, including the exercise selection and the within-exercise variables that accurately describe each exercise and characterize its execution [15,16,17,18]. More in detail, variables like the type of external resistance, movement velocity, range of motion (ROM), or performance of concentric- or eccentric-only training should be considered intrinsic to every exercise and may lead to different adaptations in performance. Such a deeper approach may be used to reduce the variability in traditional RT-induced adaptations and individualize the overall stimuli also depending on the context.

Nevertheless, most RT protocols do not properly describe the RT exercises and even a replication for practitioners leaves room for many methodological interpretations given the lack of details that characterize each exercise. Despite the complexity and relevance of the topic, no critical examination of the literature has been conducted so far, and this would allow practitioners to design RT protocols within the soccer routine better. Furthermore, this would improve the methodological approach to RT and the reproducibility of a given protocol from the laboratory to the field. Therefore, the present review intends to summarize the state of the art of the relevant literature that offers the possibility to describe the exercises planned during RT protocols to improve the jumping, sprinting, and COD ability in soccer players.

## 2. Research Strategy

Given the multitude of aspects inherent to resistance exercise training variables, to address the research question, we synthesized findings primarily from existing systematic and narrative reviews. When unavailable, we included original studies examining individual variables in isolation.

We searched the electronic databases Pubmed, Scopus, and Google Scholar for relevant articles until 31 March 2024. A further update to check for possible new articles was carried out on 15 January 2025. The keywords used were “resistance training” or “exercise” or “strength training” or “weight training” and “volume” or “load” or “range of motion” or “movement velocity” or “time under tension” or “concentric” or “eccentric” or “velocity-based training” or velocity loss” or “failure” or “exhaustion” or “attentional focus” and “squat” or “deadlift” or “quadriceps” or “hamstrings” and “soccer” or “football” and “sprint” or “jump” or “change of direction”. Only articles published in English were selected, and their bibliography was gleaned for further potentially eligible studies. Additionally, as for the original articles only, manuscripts examining the effects of an identifiable variable in isolation were included, and we excluded those where more variables were combined or the RT intervention was ambiguously described.

### 2.1. What We Know

#### 2.1.1. External Load

The external load is the amount of mechanical stimulus that the external resistance provides to the muscle–tendon complex [15]. The main quantification of the external load is based on the maximum dynamic capacity to lift a weight, i.e., the 1-repetition maximum (1-RM), and is usually expressed as %1-RM. When categorizing the external load, high load is typically ≥60% 1-RM, while low load is <60% 1-RM [19], albeit moderate load is sometimes identified as ≥60% and <80% 1-RM [20]. The literature offers some direct comparisons between different external loads when RT was included in the in-season soccer weekly routine. For example, parallel back squat was added to soccer-only training for academy players either using 80% or 90% 1-RM once a week [21]. Both loads were effective in equally improving the vertical jumping ability of the soccer-only group, while 90% squat was the only group to increase their horizontal jumps, and no change was observed in sprints [21]. Another study included half squats performed with 70% or 90% 1-RM with similar improvements in sprinting ability, while greater gains in CODs and jumps were achieved in the 90%-group [22]. Another study reported that in-season RT with a low-to-moderate (50–65% 1-RM) or null external load (0% 1-RM) was effective in improving the jumping height, with greater effects for the former [23]. Additionally, an 8-week power training intervention performing null-external-load (0% 1-RM) jump squats or loaded 30% 1RM jump squats led to similar increases in jump height and no effect on sprint time, even though CODs were improved only in the 30%-group [24]. Besides the direct comparisons using different external loads, the literature has plenty of studies that have proved the benefits of RT included in the weekly soccer routine, with a variety of external loads that can be used purposely, as summarized in [1,25]. In this regard, RT performed during the in-season period was observed as less favorable to induce improvements in jumps than RT during the pre-season, while performing RT in both periods (and so for longer durations) is shown to be effective in improving jumping performance [25]. As for sprints, RT protocols in the pre-season and in-season periods were prone to generate improvements, as well as long-term RT protocols that started during the pre-season and continued during the in-season period [25]. Practitioners should consider that the effects of moderate-to-high loads seem superior to low external loads, especially with high-level players [1,25]. As for CODs, it should be noticed that the magnitude of the external load may not be a key factor when distinguishing between moderate and high loads, and technical sessions should be dedicated to improving CODs [7]. Consequently, we suggest the inclusion of resistance training sessions with moderate-to-high external loads, with the possibility of reducing other training variables, such as the number of repetitions [26], to improve soccer-related abilities.

#### 2.1.2. Type of External Resistance

The previous section was dedicated to what is usually defined as dynamic constant external resistance, i.e., when the amount of the external load does not change across the whole movement. In this regard, a meta-analysis observed that constant loads using free weights appear more effective in improving CODs, irrespective of the load intensity [7]. However, devices different to constant load-free weights and machines may provide variable resistance across the movement, incrementing the eccentric or concentric resistance like the flywheel or the elastic resistance, respectively. Both training modalities are quite common in practice as alternatives or together with the dynamic constant external load.

Regarding the flywheel resistance, the high-velocity concentric phase generates a mechanical output that is a determinant of the eccentric load, to possibly generate an eccentric overload [27]. Incidentally, irrespective of the actual generation of the eccentric overload, the eccentric phase is performed at a relative intensity not achievable when performing traditional resistance training with constant external loads, whose load depends on the concentric capacity [28], with a lower relative resistance for the eccentric phase. Such a high-intensity eccentric phase has been suggested to mimic the braking phase of the CODs and has been recommended to be included in the soccer RT routine [29], albeit the benefits are not limited only to CODs [30,31]. A direct comparison between squat training performed once a week using either flywheel or constant 80% 1-RM appears to favor flywheel training in the in-season improvements in the CODs. CODs, with similar improvement in jumping ability and no change observed in sprints [32]. Another recent study observed similar gains in CODs comparing different in-season RT programs performed twice a week using flywheel or 60% 1-RM with advantages for the former in the jumping ability [33]. A pre-season intervention based on flywheel resistance or 30-to-70% 1-RM twice a week observed similar achievements in jumps and sprints, while CODs were not recorded [34]. Besides the direct comparisons, the literature has summarized the effectiveness of the flywheel device for improving CODs [35], as well as jumps and sprints [30]. In practice, trainers should also consider the muscle damage derived from the accentuated eccentric load using flywheel resistance, albeit this will confer protection against further similar sessions that could then be included in the in-season program [36]. In light of what the above flywheel RT may contribute to develop CODs, it could be a good alternative to traditional RT for improving jumps and sprints.

Compared to the flywheel resistance training-induced effects, the use of elastic bands in the soccer routine has been much less investigated. It should be noticed that the elastic resistance increases while the band is lengthening and decreases while shortening so that an emphasis on the concentric phase occurs. However, one of the main issues associated with elastic band training is the appropriate quantification of the mechanical load, which limits the awareness of the dosage and the possibility of managing the load appropriately. Moreover, the maximum amount of external resistance offered by an elastic band is much lower than using a traditional constant external load, so elastic bands are highly suitable for specific exercises that do not require high absolute loads or to replace part of the constant external load as often performed by practitioners [37]. A direct comparison with traditional constant resistance was performed off-season by replacing a part of this resistance with elastic bands in different exercises [38]. The results were an increase in vertical jump using the elastic but not the traditional resistance and no improvement in sprints or CODs was observed [38]. However, the load was not stated clearly, and this opens the question of what actual stimulus may have resulted from this intervention. Another direct comparison was performed pre-season using velocity-based training to match the relative intensity, although the load magnitude was not stated, and also, in this case, a part of the resistance was replaced with elastic bands [39]. Both training groups similarly increased in jumping and failed to develop sprinting ability, while only constant resistance improved the CODs [39]. We were not able to find any direct comparison, nor any other inclusion of elastic band training in the soccer routine. As such, elastic bands may be used to improve jumps although the level of evidence is quite low, while additional training for sprints and CODs is required.

#### 2.1.3. Movement Velocity

The time under tension is the sum of the tempo for each phase, whose reciprocal regarding the two dynamic phases is the movement velocity [15]. Incidentally, while a controlled movement velocity can be combined with any load, the load affects the extent of the maximal velocity achievable, i.e., greater loads correspond to slower maximal movement velocities. The anecdotal perception among practitioners is that training with high movement velocity is required to improve explosive tasks, while non-explosive movements or a slower movement velocity derived from high loads are perceived as ineffective or even detrimental. However, a meta-analysis reported that improvements in the rate of force development occur irrespective of the loads used in training and the intent to accelerate the load maximally, and are rather dependent on the training specificity [40]. While it is acknowledged that the rate of force development is assessed isometrically, this contributes to breaking the credence that absolute low velocities should be avoided. That said, the literature reports that increasing the duration of the eccentric phase from 2 s to 4 s did not add any advantage in increases in jump height, while both protocols did not improve CODs [41]. Moreover, elite soccer players underwent an in-season RT with the eccentric phase performed either in 1 s or 4 s [42]. The outcomes revealed that both durations similarly improved sprints, but the longer duration was detrimental to the jumping ability [42]. No other direct comparison was retrieved, so it seems that performing controlled movements in a normal or rapid manner is more effective than intentionally slowing them.

In recent years, the velocity-based training (VBT) method has gained considerable attention among practitioners since it emphasizes the concept of maximal movement velocity (for the concentric phase) so that each repetition is performed with the intent to maximally accelerate the load concentrically [43]. The literature has plenty of studies that investigated the effects of VBT, and different meta-analyses established its effectiveness for improving muscle strength and power [44,45,46], as well as jumping and sprinting ability [45,47]. Although a sound basis theoretically underlines the superior explosive training/task specificity, when directly compared to traditional RT, which does not focus on maximal velocity, VBT was not more effective in increasing power and sprint speed [48]. Moreover, another meta-analysis reported no superiority of VBT compared to traditional RT in improving strength, jumps, and sprints [49]. A further meta-analysis observed a similarity between velocity-based and traditional RT in improving strength, jumping, linear sprinting, and CODs [50]. Mechanistically, it should be recalled that power is a combination of force and velocity, and focusing solely on the movement velocity does not guarantee power gains. Consequently, the optimization of the training should be based on different load/velocity pairings to maximize power. In view of the above, performing RT at maximal velocity is not a prerogative of effectiveness for improving such soccer-related abilities. This implies that traditional RT performed with controlled movement velocity is as effective as VBT in improving jumps, sprints, and CODs. However, intentionally over-slowing the movement velocity in traditional RT with the purpose of increasing muscle engagement does not provide any advantage.

#### 2.1.4. ROM

Another point of debate in RT, especially regarding performance, is the ROM to be used in the exercises selected. In particular, the squat is largely included in many training cycles, and the anecdotical opinion is that the resistance exercise should reflect the task specificity to maximize the transfer, especially in jumping ability [51], so that a half squat mimics the jump loading and is expected to be more effective than other squatting depths. The literature comparing different squat depths incorporated in the in-season RT soccer routine is lacking, albeit some results can be extrapolated from different contexts. The effects of full, parallel, or half squats with a relative load of 60–80% 1-RM on jumps and sprints were examined in resistance-trained men after a 10-week cycle [52]. The outcomes showed an increasing superiority from half to full squat concerning the gains in jump height, while the 20 m sprint time decreased only after the full and parallel but not half squat [52]. Another study tested full vs. 60° (0° full extension) knee flexion squats performed three times a week for 12 weeks, with a load ranging from 10-RM to 3-RM for both training groups [53]. After the training period, both groups improved the countermovement jump, while only the full squat improved the squat jump height [53]. Still, full squat performed with 10-RM to 2-RM twice a week for 12 weeks increased the countermovement and squat jump height, while quarter squat did not [54]. These findings challenge the assumption that partial ROM exercises are more specific or superior for jump performance, suggesting that full ROM may offer greater benefits. Importantly, the previous studies used the same relative load for comparing full vs. partial squat, which means that the absolute load was greater for the partial squat given the shorter displacement. Notwithstanding, ROM appeared greater than load as a moderating factor in these cases. No information was retrieved for CODs, and further investigation is needed.

### 2.2. What We Do Not Know

#### 2.2.1. Eccentric-Only vs. Concentric-Only vs. Traditional Eccentric–Concentric Training

While traditional RT is based on the execution of both the eccentric and concentric phase irrespective of the constant or variable load as examined in the previous section, another possibility is to perform the concentric or the eccentric phase of a given exercise alone or to perform exercises that place a greater emphasis on one of the two phases, such as Nordic hamstring exercises for the eccentric and deadlifts (releasing barbells once in the upper endpoint) for the concentric phase. Previous studies highlighted the superiority of volume-equated eccentric-only RT over concentric-only and traditional eccentric–concentric RT to increase and especially retain strength after detraining [55,56,57,58], with a greater neural transfer in the contralateral non-trained limb [59,60,61]. Additionally, eccentric-based RT has been advocated to reduce hamstring strain injury risk [62], and it is largely used in soccer conditioning purposely [63].

As regards jumps, sprints, and CODs, a previous systematic review indicated that eccentric-only training like Nordic hamstring exercises may be beneficial to improve sprints and jumps in young soccer players and adults, but no indication was provided for CODs [64]. A subsequent systematic review added information about the effectiveness of eccentric-only training to improve the COD ability, confirming it for the jumping and sprinting abilities [65]. We could not find any study that directly compared the effects of eccentric- vs. concentric-only resistance training on performance, so any indication here would be speculative. We would just like to remind that eccentric-only training may require long initial between-session recovery [36,66,67,68,69,70,71], so its scheduling into the soccer routine should be programmed carefully.

#### 2.2.2. External vs. Internal Focus

Attentional focus is a cognitive resource used to potentially implement neuromuscular processes while performing a given task [72,73]. More specifically, external focus reflects the attention paid to the task while internal focus is on a muscle group that is involved in the task [72,73]. Previous studies have indeed reported increases in activation with an internal focus in various muscle groups for a given load [74,75,76,77], with possible advantages in long-term hypertrophic but not strength adaptations [78]. However, the fact that more neuromuscular coordination is needed for the same task raises questions about the usefulness of the internal focus as a source of decreased movement economy [79], especially considering that the force expression does not change or may even decrease [80,81]. In this view, complex tasks like jumps, sprints, or CODs are shown to be acutely impaired by an internal focus [72,73], even though no long-term comparison has been made. Therefore, although no specific study has been conducted so far, the suggestion is to prefer external over internal focus while practicing RT exercises.

#### 2.2.3. Sets to Failure/Non-Failure

Performing sets to failure implies that the number of repetitions within each set comes from exhaustion and is not fixed a priori, with the chance to increase the total number of repetitions performed with a given load and number of sets [15]. Possible advantages from failure sets involve hypertrophic gains, while non-failure seems more involved in optimizing strength, even though when the total number of repetitions remains constant, the differences tend to disappear [82,83]. When comparing the long-term effects on soccer-related abilities, an 8-week intervention to failure vs. non-failure did not show any difference in the improvement in jumping, sprinting, and COD ability [84], while another study reported greater rises in jump height and rate of force development after 10 weeks in non-failure vs. failure RT [85]. No further direct comparison was found, with the literature indicating that reaching failure does not seem necessary to improve performance.

Another method that seeks to avoid failure is velocity loss training, a variant of VBT that interrupts each set when the movement velocity falls below a given threshold fixed a priori [49]. The rationale is to avoid a fatigued status that would impair the capacity to maximally accelerate the load and induce a meaningful drop in the movement concentric velocity. In this sense, different velocity loss thresholds may be fixed, which are the maximum movement velocities recorded during the set supposedly occurring during the very first set of repetitions in a non-fatigued state, with possible moderating effects on performance. For example, fixing the threshold at −15% vs. −30% in the in-season weekly routine of professional soccer players resulted in greater achievements in jump height albeit no group improved in sprints [86]. Overall, both thresholds below and above −20% velocity loss appear effective in improving jumps and sprints [49], even though low-velocity loss thresholds (<−15%) seem recommended [87]. No further information is available for CODs.

Besides the comparison of failure vs. non-failure long-term effectiveness, there are some practical considerations. First, sets to failure lead to fatigue, and the technique warranted for each repetition may be somehow affected. Second, some exercises have a high risk of injury when performed to failure (e.g., squat) while others may be performed with a certain degree of safety (e.g., deadlift), with single-joint exercises appearing anyway preferable in a failure context [88]. Third, failure usually requires more time for recovery [88]. A recent study indeed compared the acute effects of failure vs. non-failure equated for volume with the time to recover to baseline [89]. The non-failure group experienced a fall in power and jumps 15 s after and recovered after 10 min, while the failure group experienced detrimental effects for up to 30 min [89]. Fourth, and strictly connected with the previous point, the recovery after the training session may be considerably slowed, with possible repercussions in terms of injury risk [88]. Fifth, training to failure is perceived as more fatiguing [90] and is based on more repetitions [86], and this may undercut adherence to training. On these bases, non-failure training is overall recommended and there is no apparent reason to include failure in the in-season RT program to improve jumps, sprints, and CODs.

#### 2.2.4. Total Number of Repetitions (Volume)

The total number of repetitions (i.e., sets × repetitions) can be used as a quantification of the volume for a given resistance exercise, provided that all variables have been fixed [15]. While this refers to a single session, it should also be noticed that the volume may refer to the whole number of repetitions performed in each training cycle, so that incrementing the frequency of a given session also results in increments in volume. A further way to raise the volume is incrementing the training period in terms of weeks. It is known that increasing RT volume leads to greater hypertrophic gains [90] but has little effect on strength [91], with a minimal dose required to promote strength increments in resistance-trained men [92]. Interestingly, augmenting frequency results in greater strength gains because of the greater volume, but volume-equated frequencies have similar effects [93]. As regards performance, a previous meta-analysis summarized the dose–response effect of RT in youth athletes [94]. In the first instance, longer periods (>23 weeks) appear more beneficial for improving CODs, while intermediate periods (9 to 12 weeks) seem to optimize the jumping ability [94]. Incrementing the number of sets and sessions seems to induce similar adaptations, so incrementing the training volume does not provide any advantage [94]. However, a following study investigated the effects of an RT session performed once or twice per week, i.e., doubling the volume, for seven weeks on jumps, sprints, and CODs [95]. After the intervention, the jumping ability improved similarly, while sprints and CODs improved in the two-session more than in the one-session group [95]. Taken together, the dose–response relationship between RT and improvements in soccer-related abilities is not always linear, with CODs especially needing greater RT volume than jumps and sprints to be improved. This invites practitioners to consider the configuration of RT protocols within the soccer routine as dependent on the specific purpose since greater volume requires a longer time to recover. Consequently, RT performed with the intent to improve jumps and sprints may coexist more easily with other drills, while RT to improve CODs requires greater emphasis and may occupy a greater part of the soccer routine in academy players. As for adults and specifically for CODs, factors like training duration, frequency, and total number of sessions do not play a role, so that resistance training routine may be designed depending on the overall purposes of the training session and cycle [7].

#### 2.2.5. Inter-Set Rest

Inter-set rest is the time between two sets of the same exercises or between two different exercises [15]. Before examining the rest duration, it should be noted that recovery can be passive or active, and some active strategies may enhance acutely the force and power exertion [96]. For example, static stretching of the antagonist muscles or dynamic stretching of the agonists, cooling, aerobic exercise, and vibration might be effective methods to optimize recovery and improve acute RT performance [96]. However, while it is conceded that we do not know the long-term effectiveness, it should also be acknowledged that performing active recovery strategies in soccer practice could not be easy to assess. As for passive recovery, inter-set rest longer than 2 min has been shown to favor muscular strength [97]. Similarly, at least 2 min recovery is suggested to optimize the jumping, sprinting, and COD ability [94]. In this light, avoiding acute fatigue is recommended to preserve the warranted exercise technique and not incur acute impairments in force-generating capacity, especially rapid force exertion [98]. Notwithstanding, direct comparisons are not available, and this rationale needs to be verified.

#### 2.2.6. Exercise Selection

While the previous variables can be referred to any resistance exercise, the choice of the exercise to be included in the RT soccer routine is another relevant point to be addressed since each exercise has its technique and prime movers and could consequently provide the athletes with specific neural and mechanical stimuli. Overall, the RT exercises in soccer are chosen to stimulate mainly the lower-limb muscles, and although these are numerous, some of them are recurrent both in the literature and in practice, such as squats or deadlifts and their variations, and, more recently, hip thrusts. In particular, hip thrusts intend to accentuate the role of the hips, with different characteristics compared to squats and deadlifts [99]. Though the literature is mostly focused on the effects of one or more exercises included in the RT routine, direct comparisons have been scarcely conducted. However, front squat and hip thrusts were equally effective in improving jumping ability after a 6-week intervention, while 10 m and 20 m sprint time decreased in the hip thrust but not the front squat group [100]. Another study observed similar increases in jump height and decreases in 10 m and 20 m sprint time after a hip thrust vs. back squat 7-week intervention performed off-season by adolescent female soccer players [101]. No further direct comparison was found; hence, no information is available for CODs. In this light, no exercise showed clear superiority in improving performance, so RT should be planned to include one or more exercises or variations.

## 3. Limitations

This is the first attempt to examine the effects of the independent variables referring to conventional exercise when including RT in the soccer routine. While none of the variables is per se a novelty, sports scientists and practitioners have often based RT incorporation on the total number of repetitions and the load. Consequently, we were not able to find a good level of evidence in support of each variable and this represents the main limitation of the present review. Future studies should systematically investigate the effects of the manipulation of each variable to provide more robust indications. Furthermore, a detailed description of the execution of a given exercise would improve the methodological approach to RT and would also favor the reproducibility of the training session. Another point is that plyometric training was not included. We acknowledge the effectiveness of plyometric training for improving jumps, sprints, and CODs [2,11,12,13,102]. However, most of the independent variables are hard to quantify so we suggest considering it as a standalone RT modality rather than derived from the combination of conventional RT exercise variables.

## 4. Conclusions

Designing RT in soccer requires many more details than just prescribing an exercise with sets, repetitions, and loads. The present conceptual review was designed to summarize the literature examining how the manipulation of different independent variables in RT exercises may affect the jumping, sprinting, and COD ability of soccer players.

As regards jumps, RT appears quite effective. Moderate-to-high loads, movements performed at full ROM, non-failure sets, and a moderate total number of repetitions may be suggested, while the type of external resistance like constant-load, flywheel resistance, or the use of elastic bands, as well as different movement velocities and the exercises selected, appear equally effective. As for sprints, the effects of RT appear relatively low. With this in mind, moderate-to-high loads, constant-load or flywheel resistance but not elastic resistance, movements performed at full ROM, non-failure sets, and a moderate-to-high total number of repetitions might be more effective, while a wide range of movement velocities and exercises could be chosen. As for CODs, there are some independent variables with clear directions, while others need further investigation. Moderate-to-high loads, flywheel resistance more than constant-load and elastic resistance, non-failure sets, and a moderate-to-high total number of repetitions may be suggested, while a range of movement velocity and exercises could be selected, and we did not find any evidence concerning the suggested ROM. It should be noticed that the impact of the attentional focus has not been investigated, albeit the theoretical basis suggests that the internal focus should be avoided in favor of an external focus. Similarly, the possibility of performing concentric-only or eccentric-only instead of traditional eccentric–concentric exercises requires investigation concerning the effects on performance. Lastly, inter-set rest longer than 2 min is suggested, albeit more robust evidence is needed.

Table 1 summarizes the main points.

Practitioners have now more specific information for prescribing RT exercises in soccer routines and manipulating the independent variables depending on the specific purpose of the session. Interestingly, movement velocity does not play a decisive role in developing jumps, sprints, and CODs since a wide spectrum of movement velocities that do not intentionally over-slow the exercise execution can be selected. Moreover, ROM may impact jumps and sprints (but no information is available on COD), but contrary to what is believed, i.e., full ROM might be preferable.

Lastly, some considerations should be made to include overall RT in the weekly periodization. Indeed, RT may often induce an interference effect towards the rest of the training session due to muscular fatigue [103], or the following days due to muscle damage induced by the active muscle lengthening [67]. This implies that RT has to be carefully done, especially in the congested in-season period, so that previous accustoming phases should have been performed before, possibly pre-season, from the official matches to provide time to fully recover. Additionally, the magnitude of the RT-induced adaptations may also depend on the player’s experience, strength level, and training history. Consequently, the periodization of the RT dose and manipulation of the aforementioned variables should take all these aspects into account.

In conclusion, it should be remembered that RT may help to develop performance, but specific drills are needed to improve jumps, sprints, and CODs.

## Figures and Tables

**Table 1:** A summary of the influence of the resistance training variables on jumps, sprints, and changes of direction.

Influence of Resistance Training Variables on Jumps, Sprints and Changes of Direction (CODs)		
Resistance Training Variable	What Is Known	What Is Not Known
Magnitude external load	Moderate-to-high > low loads	Short-term recovery should be considered after high vs low loads
Type external resistance	Flywheel and constant external loads are equally effective for jumps and sprints. Flywheel is possibly better than constant load for CODs.	Elastic training as an alternative method for sprints and CODs.
Movement velocity	Movement velocity does not affect gains in jumps, sprints, and CODs.	Over-slowing movement velocity (>4 s per phase) should be avoided, although not completely proven.
Range of motion (ROM)	Full > partial ROM for jumps.	No sufficient information for ROM and sprints and CODs.
Eccentric vs. concentric vs. traditional eccentric/concentric training	Eccentric training is effective in improving jumps, sprints, and CODs. Eccentric training requires long initial recovery.	No direct comparison, so it is not clear if one methodology is more, less, or equally effective.
External internal focus	External focus should equalize or increase strength during a movement. External focus suggested for complex tasks.	No direct long-term comparison, albeit external focus has more favorable theoretical bases.
Failure/Non-failure	Velocity loss thresholds > 15% to improve jumps and sprints.	Few information indicates non-failure > failure. No information about velocity loss thresholds and CODs.
Total number of repetitions (N)	A wide range of N could be selected to improve jumps, sprints, and CODs. Higher N is needed to improve CODs than jumps and sprints in young athletes.	N may not affect COD in adults. No information about the effects of N of jumps and sprints in adults.
Inter-set rest	Longer durations may be suggested since it is more effective for increasing strength.	No direct comparison between longer/shorter durations available.
Exercise selection	Squat variations, hip thrusts, and deadlift improve sprints equally.	No exercise superiority can be claimed. No information for jumps and COD.
